# Evolution of Primate Sense of Smell and Full Trichromatic Color Vision

**DOI:** 10.1371/journal.pbio.0020033

**Published:** 2004-01-20

Conventional wisdom says that people deficient in one sense—such as vision or hearing—often acquire heightened acuity in another. And some studies support this notion by showing that areas of the brain known to control vision can respond to other forms of sensory stimuli in persons without sight. These adjustments, of course, take place over the lifetime of an individual. Now it appears that similar adjustments may occur over evolutionary time. Investigating the deterioration of olfactory receptor (OR) genes in primates, Yoav Gilad and his colleagues at the Max Planck Institute for Evolutionary Anthropology in Germany and the Weizmann Institute in Israel found a correlation between the loss of OR genes and the acquisition of full trichromatic color vision.

OR genes—the molecular basis for the sense of smell—form the largest gene superfamily in mammalian genomes. But a high percentage of these genes are “pseudogenes,” DNA sequences that are remnants of genes that are no longer functional. Following an evolutionary “use-it-or-lose-it” rule, pseudogenes tend to evolve in larger gene families where there's no selective advantage in having, say, 100 versus 120 genes. While humans, nonhuman primates, and mice have roughly the same number of OR genes, in humans a much higher percentage of these are pseudogenes, at 60%, while nonhuman apes have about 30%, and the mouse has about 20%. Reliance on the sense of smell, it appears, decreases for animals that develop a dependence on other senses, such as hearing or sight, to survive. In characterizing this high proportion of pseudogenes, Yoav Gilad et al. asked: Is this characteristic of all primates? If not, at what point in primate evolution did the increase occur?

Looking at 19 primate species—including one human, four apes, six Old World monkeys, seven New World monkeys, and one prosimian—Gilad et al. randomly sequenced 100 distinct OR genes from each of the species. The team found that Old World monkeys had roughly the same percentage of OR pseudogenes as nonhuman apes, but a much higher percentage than New World monkeys—except for one, the howler monkey. The percentage of OR pseudogenes in the howler monkey was much closer to that seen in the Old World monkeys and apes than in its New World cousins. The sense of smell, it appears, deteriorated both in the ape and Old World monkey lineage and in the howler monkey lineage. Since Old World monkeys, apes, and the howler monkey do not share an exclusive common ancestor, this deterioration must have evolved independently in both groups. Surprisingly, howler monkeys share another sensory feature with apes and Old World monkeys: trichromatic color vision.

In trichromatic color vision, three retinal protein pigments, called opsins, absorb various wavelengths of light, which the brain processes to produce full-color images. Apes and Old World monkeys carry three opsin genes, and most New World monkeys carry only two, though females can sometimes have three. Only howler monkeys routinely have three genes occurring in both sexes. Thus, full trichromatic vision evolved twice in primates—once in the common ancestor of apes and Old World monkeys, about 23 million years ago, and once in the howler monkey lineage, about 7–16 million years ago. The evolution of color vision, the authors propose, coincided with a growing complement of OR pseudogenes and a deterioration of the sense of smell. Gilad et al. suggest that investigating the types of visual cues required for finding food may shed light on the nature of this connection.

**Figure:**
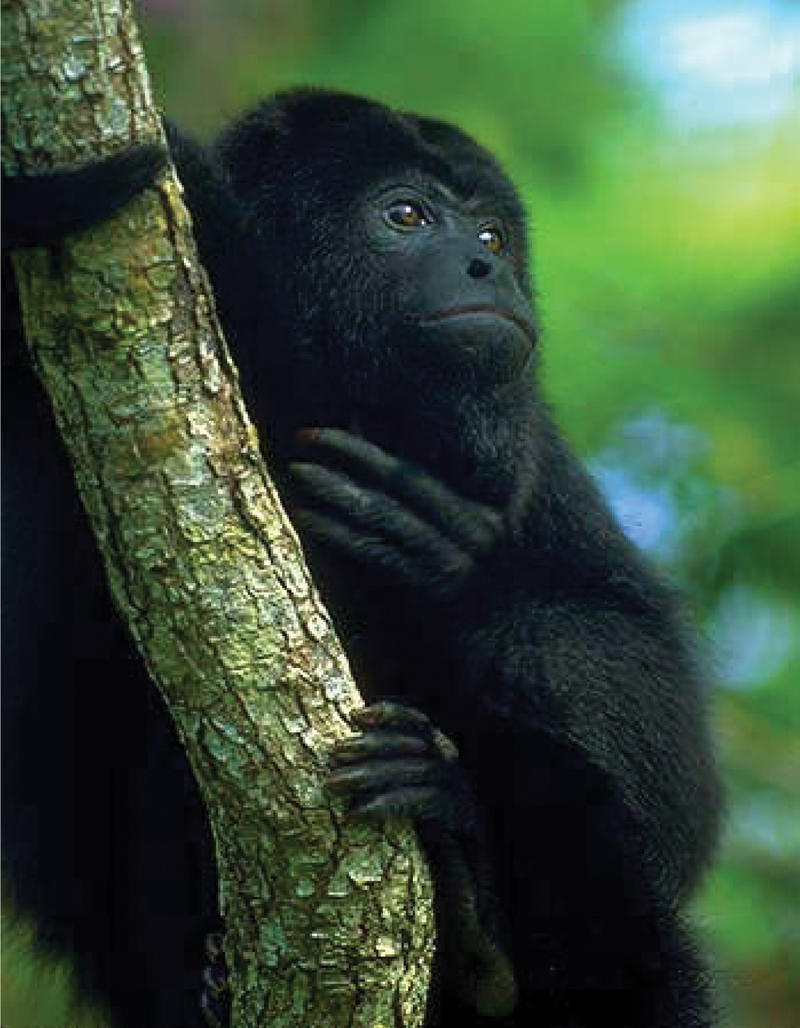
Howler monkey

